# Early Economic Model of Extracorporeal Photopheresis and Other Treatments of Chronic Lung Allograft Dysfunction After Lung Transplantation

**DOI:** 10.36469/001c.163450

**Published:** 2026-09-22

**Authors:** Stephen Rice, Diarmuid Coughlan, Luke Vale, Carole Cohen, Christian Benden, David Bennett, Mark Greer, Jasvir Parmar, Adrien Tissot, Robin Vos, Myriam A. Perez, Martin Carby, Richard Thompson, Karthik Santhanakrishnan, Andrew J. Fisher

**Affiliations:** 1 Population Health Sciences Institute Newcastle University, UK; 2 Centre for Reviews and Dissemination, University of York, UK; 3 Axispharma Ltd on behalf of Therakos Ltd; 4 University of Zurich Medical Faculty, Zurich, Switzerland; 5 University Hospital of Siena, Siena, Italy; 6 Hannover Medical School, Hannover, Germany; 7 Royal Papworth Hospital, Cambridge, UK; 8 Service de Pneumologie, l’institut du thorax, Center for Research in Transplantation and Translational Immunology Nantes Université, CHU Nantes, INSERM, Nantes, France; 9 Department of Respiratory Diseases, Lung Transplant and Respiratory Intermediate Care Unit University Hospitals Leuven, Belgium; 10 Hospital Universitario Puerta de Hierro Majadahond, Madrid, Spain; 11 Royal Brompton and Harefield Hospitals, London, UK; 12 University Hospitals Birmingham, UK; 13 Manchester University Foundation Trust, Wythenshawe Hospital, UK; 14 Translational and Clinical Research Institute Newcastle University, UK

**Keywords:** cost-effectiveness analysis, lung transplant, chronic lung allograft dysfunction, decision model, extracorporeal photopheresis, Markov model

## Abstract

**Background:**

Extracorporeal photopheresis (ECP) is a technology to treat chronic lung allograft dysfunction (CLAD) following lung transplantation. CLAD leads to significantly higher mortality rates.

**Objective:**

The objective of this study was to develop an early economic model that can evaluate the potential cost-effectiveness of treatments for patients with a confirmed diagnosis of CLAD and to evaluate a plausible cost-effectiveness range for ECP.

**Methods:**

The literature was reviewed and clinical experts were consulted to develop a Markov model with change in percent forced expiratory volume in 1 second (%FEV1) states, defining rates of lung function decline. The literature and clinical experts were also consulted to determine plausible transition probabilities between %FEV1 states and resource use. Probabilities of dying were estimated by calibrating the model to published survival curves. Treatment response was obtained from cohort studies. EQ-5D values and unit costs were obtained from published sources.

**Results:**

In the base case analysis, the incremental cost-effectiveness ratio (ICER) estimates for ECP ranged from £33 343/QALY to £88 278/QALY. This does not account for risk of bias in the implicit relative risk estimate in the model or for scenario combinations. The ICER was lower in the bronchiolitis obliterans syndrome population than in the restrictive allograft syndrome population and in earlier CLAD stages. The ICER was lower if ECP was provided for a course of treatment and there was a persistent protective effect from lung function decline after cessation of treatment.

**Conclusion:**

There was very little quality evidence to inform the decision model. The results included a wide range of ICERs. Key determinants include the therapeutic benefit of ECP after treatment cessation, response rates with standard care, and CLAD stage mortality rates.The economic model can be updated as new evidence emerges from ongoing clinical trials. For the National Institute of Health and Care Excellence in England, ECP may qualify as a highly specialized technology, for which the cost-effectiveness threshold may be £100 000/QALY or higher.

## BACKGROUND

In 2023, approximately 6800 people received a lung transplant worldwide, with almost half of these in the Americas (mainly the United States).^1^ Long-term survival after lung transplantation is limited compared with other solid organ transplants,^2^ with survival for adults following transplantation reported as 56.2% at 5 years in the United Kingdom^3^ and 63% in France.^4^ The main cause of the limited survival is development of progressive immune-mediated damage to the transplanted lung. There are several ways by which this damage could occur, but collectively they can be described by the term chronic lung allograft dysfunction (CLAD), which is often referred to as a chronic rejection.^5^ There are 5 CLAD stages, each defined by the forced expiratory volume in the first second (FEV_1_) as a percentage of a baseline FEV_1_ post-transplantation.

Currently, treatment options for CLAD are limited, as there are no treatments that can reliably halt or reverse CLAD.^5,6^ Internationally, the standard of care is mainly supportive, aiming to control symptoms and provide psychological support as quality of life falls and activities of daily living become increasingly limited. For those with the most severe CLAD, the only option is lung retransplantation, but for many even this option is not possible.^7^

Extracorporeal photopheresis (ECP) has been reported to offer clinical benefit by slowing or halting CLAD progression in selected patients.^5^ A small amount of blood from the patient is removed, and white blood cells are exposed to a photosensitizing agent (methoxsalen) and ultraviolet A light, which causes them to die, very likely of apoptosis. The treated white blood cells are then returned to the patient, where they exert an effect on other circulating immune cells. ECP is currently widely used in conditions such as graft-vs-host disease after hematopoietic stem cell transplantation and cutaneous T-cell lymphoma and is generally considered safe and well tolerated.^8^

Nonrandomized evidence suggests that ECP may be beneficial to selected patients with CLAD in terms of response and survival.^9^ More evidence is needed, and there are ongoing trials on the use of ECP that are seeking to address how ECP may be used in the care pathway as a therapy for CLAD. The currently available data is sparse with respect to lung function decline rates and trajectories, mortality rates, health-related quality of life (HRQoL), and healthcare costs associated with CLAD stages for standard care and ECP, and these trials should provide relevant evidence.

We are unaware of any published economic evaluations of treatments for CLAD. Economic evaluation of treatments for CLAD is challenging but also crucial to understand, as CLAD is one of the key causes of post-lung-transplant morbidity and the high mortality rate experience by those who receive lung transplants and no satisfactory treatments are available.

Given the possible promise that ECP may be an effective treatment option for CLAD, the objective of this study was to develop an early economic decision model^10^ to evaluate plausible ranges of cost-effectiveness of ECP vs standard care in CLAD patients in the UK, to identify how effective ECP must be compared with standard care for it to be cost-effective, and to identify the model assumptions and parameters that have the greatest impact on the ICER results. The latter will help guide further research and evidence generation from future studies. A final aim is to provide a framework to evaluate the cost-effectiveness of ECP in other countries wherever and whenever more complete data is made available. As this is a fast-moving area of clinical research, our model would also be suitable for adaption to evaluate other, currently unspecified, treatments for CLAD.

## METHODS

An early economic evaluation decision-analytic model was designed to provide the cost-effectiveness of any intervention available to treat CLAD following lung transplantation, where there is evidence of treatment response over the first 6 months. The model has currently been populated with data for the UK and used to assess plausible cost-effectiveness ranges of ECP for CLAD treatment. A set of base-case parameter values and assumptions was established to achieve this and to enable sensitivity and scenario analyses to be conducted. Methods were consistent with National Institute of Health and Care Excellence (NICE) method guidance (see **Supplementary Material**); while specific to England, this methods guidance gives a recognized framework for economic evaluation internationally.^11^ The model can therefore be adapted to any country, providing local data is available.

### Model Design

To develop the structure of the decision-analytic model, we first established an understanding of CLAD diagnosis, management, and treatment using the E-CLAD UK trial protocol^8,12^ and a targeted literature review. We then held a series of follow-up semistructured online interviews of the same clinical experts (UK and international) who responded to a questionnaire on the clinical factors affecting HRQoL, costs and mortality. The interviews were conducted by an experienced health service researcher. A panel discussion was then held to discuss each of the issues listed in the online interviews along with the relevant evidence identified in the literature. The rate of lung function decline was identified as the key clinical outcome determining HRQoL, costs, and clinical decision-making. A CLAD-specific treatment care pathway was then described, which was used to develop a core Markov model (see **Supplementary Material**).

**Figure 1.**
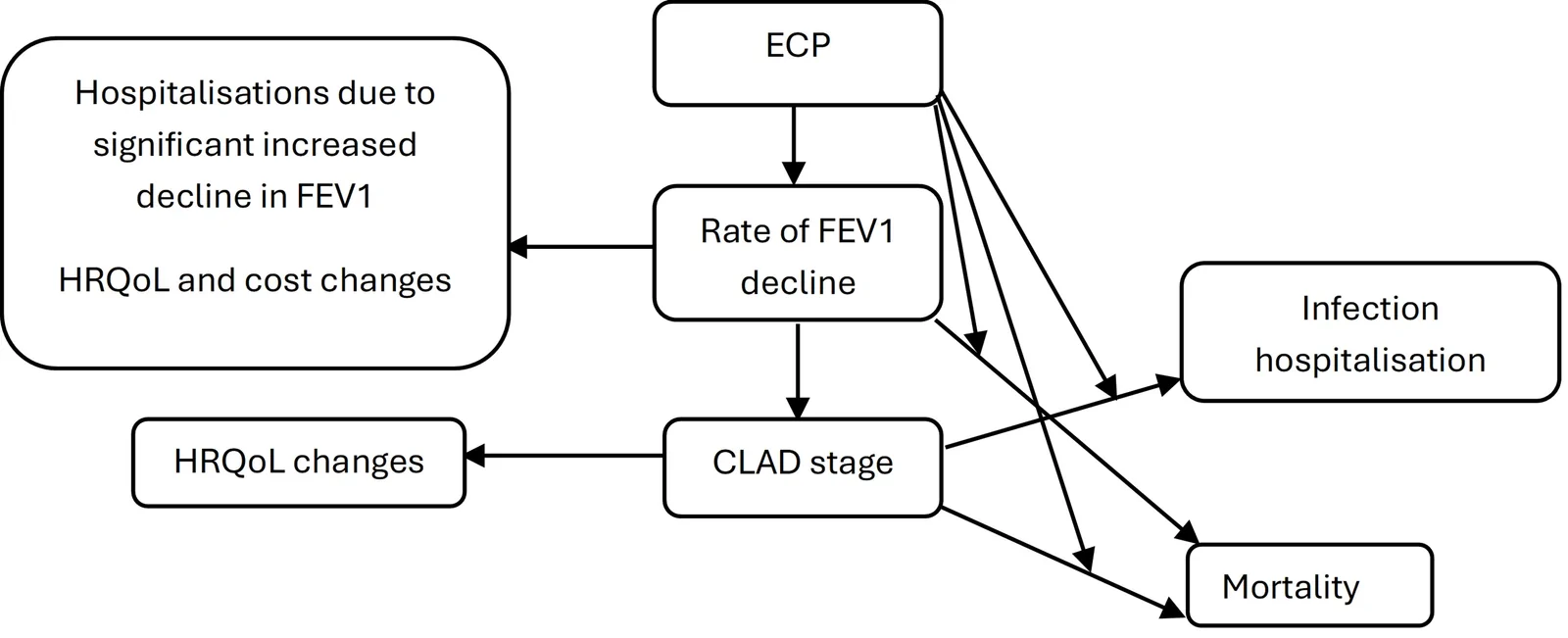
Model Influence Diagram for the Effect of ECP on Cost and Health Outcomes Abbreviations: CLAD, chronic lung allograft dysfunction; ECP, extracorporeal photopheresis; FEV_1_, forced expiratory volume in 1 second; HRQoL, health-related quality of life.

A Markov model was chosen to track the number of patients in key health states over time as different mortality rates, quality of life and healthcare resource use are associated with these states. **Figure 1** presents how treatment may affect these outcomes in the model. Treatment affects the rate of decline in FEV_1_, which influences CLAD stage progression. Both the rate of decline in FEV_1_ and CLAD stage affect HRQoL and mortality. Healthcare resource use is primarily influenced by the rate of decline in FEV_1_, but CLAD stage affects the rate of hospitalization due to infection. Treatment such as ECP may affect mortality rates and hospitalization rates. There are 5 lung function decline states in the model: slight improvement, no change, mild decline, moderate decline, and steep decline.

Mortality rates, costs and HRQoL were assumed to vary by rate of decline in FEV_1_ and CLAD stage. The model allows for the possibility that an intervention may affect hospitalizations due to infection by CLAD stage and mortality rates by CLAD stage and by rate of decline in FEV_1_. Mortality associated with an infection was included within mortality estimates within each combined state of rate of decline in FEV_1_ and CLAD stage. The cost of hospitalization associated with infection was included in the model. An intervention affects outcomes in the model by increasing the percentage of patients who have a mild rate of decline or no decline in FEV_1_ and decreasing the percentage of patients with a moderate or steep decline in FEV_1_.

### Key Model Assumptions

All people with CLAD were assumed to be in an unstable FEV_1_ decline category (>10% decline over 6 months) at entry to the model. Response to treatment was assumed to move patients to a stable FEV_1_ decline category in the first model cycle.

All the FEV_1_ decline states were replicated in the model so that there are a set of states for patients who respond to treatment within 6 months and for patients who do not respond to treatment. This allows for different transition probabilities between FEV_1_ decline categories, and therefore different lung function trajectories; specifically, patients who do not respond would decline at a faster rate on average than those who do respond to treatment. The likelihood of transitioning from one rate of decline in FEV_1_ to another depends on the response rate of the intervention for as long as the intervention is assumed to have continued effectiveness post cessation of treatment. If a patient initially responds to treatment but the rate of decline in FEV_1_ subsequently increases to a steep decline, it is assumed that the intervention is no longer effective.

In the base case, ECP is provided for those with confirmed CLAD for 12 months only. A patient receives 9 cycles (2 treatments per cycle on consecutive days) of ECP in the first 6 months. They then receive 6 cycles of ECP in the second 6 months if they responded to treatment after the first 6 months. A treatment effect was assumed to continue after cessation of ECP for 10 years with the effect waning linearly to zero over that period.

### Model Probabilities

No published evidence was identified that could have been used to model lung function decline trajectories and transitions between lung function decline rates for standard care. Consequently, a clinical expert elicitation exercise was undertaken. Transition probabilities were derived and then adjusted following comments from clinical experts. Further details on how transition probabilities were derived are in the **Supplementary Material**. The model allows for transition probabilities to change over time should evidence become available.

No published evidence on the probability of dying for each FEV_1_ decline/CLAD stage were identified; consequently, these were imputed using the model predictions of lung function decline trajectories and target survival curves. The sources for the survival curves and the mortality probabilities are presented in the supplementary appendix. The model predicted survival curves for ECP and standard care and the curves of the percentage of patients with stable lung function, unstable lung function and death for both ECP and standard care are presented in the **Supplementary Material**.

No high-quality evidence on the effectiveness of ECP vs standard care was identified. Instead, response rates for ECP and standard care were taken from published cohort studies.^9,13^ Upper and lower 95% confidence interval (CI) limits from these sources were used as high and low estimates of response for each intervention. Including the response rates for each intervention in the model means that there is an implicit estimate of relative effectiveness of ECP vs standard care (2.63). This estimate has a high risk of bias.

For CLAD, the response rate for standard care (0.272, 95% CI: 0.219- 0.339) was obtained from Todd et al (2019).^13^ For ECP the response rate was (0.617, 95% CI: 0.579 to 0.659) obtained from Benazzo et al.^9^ The response rates for brochiolitis obliterans syndrome and restrictive allograft syndrome were derived from data reported by Benazzo et al^9^ for ECP and in Todd et al^13^ for standard care (see **Supplementary Material**).

Rates of hospitalization are assumed to increase by CLAD stage. The annual probability of hospitalization for standard care was estimated by a clinical expert (A.F.). Hospitalization was also assumed to occur if the rate of decline in FEV_1_ significantly increased.

A summary of the sources of the model parameter data is presented in **Table 1**.

**Table 1.** Summary of Parameters and Data Sources

**Parameter**	**Source**
CLAD baseline lung function decline risk probabilities (standard care)	Different lung function decline trajectories and proportions translated in probabilities by modeler. Further adjustment based on expert validation. Initial 6-month response based on Todd (2019).^13^
BOS and RAS baseline lung function decline risk probabilities (standard care)	Response probabilities calculated as weighted averages based on Todd (2019).^13^ RRs of BOS and RAS vs CLAD were calculated and multiplied with the CLAD lung function decline risk probabilities.
ECP CLAD response probabilities lung function decline risk probabilities	Response probability based on Benazzo (2024).^9^ RR of ECP vs standard care was calculated and multiplied with the CLAD lung function decline risk probabilities (standard care).
ECP BOS and RAS lung function decline risk probabilities	Response probabilities calculated as weighted averages based on Benazzo (2024).^9^ RRs of ECP vs standard care for BOS and RAS were calculated and multiplied with the BOS and RAS lung function decline risk probabilities.
CLAD mortality rates	Base case mortality rates are in-between Todd (2014)^14^ and Nykanen (2020)^15^ mortality rates. Higher mortality scenario based on Todd (2014).^14^ Lower mortality scenario based on Nykanen (2020).^15^ Further scenarios: in-between Gautschi (2024)^16^ and Benazzo (2024)^9^ mortality rates. Higher mortality scenario based on Gautschi (2024).^16^ Lower mortality scenario based on Benazzo (2024).^9^
Hospitalization rates	Standard care: expert opinion ECP: Assumption 80% of standard care rates in base case
Utilities	Base case values for CLAD stages and FEV_1_ decline rates based on EQ-5D values for FEV_1_ levels and HADS anxiety reported in Esquinas et al (2020).^17^ Alternative sets of utilities were based on Gold criteria utilities reported in Kim et al^18^ capped at 0.8 (York)^19^; CLAD 1/steep decline utility was based on the EQ-5D-5L utility state 43324 (0.3) and the CLAD 4/steep decline utility was based the EQ-5D-5L utility state 43434 (0.215); and combinations of these.
Disutility of ECP	Assumption of utility reduced by 0.1 for 1/2 day per session
Medication, diagnostic test, staff resources	E-CLAD UK trial protocol and expert opinion
Unit costs	ECP: From Mallinckrodt Staff: From PSSRU^20^ Medication: From BNF^21^ and eMIT^22^ Diagnostic tests: From Newcastle University Hospital Trust costing sheet

Abbreviations: BNF, British National Formulary; BOS, bronchiolitis obliterans syndrome; CLAD, chronic lung allograft dysfunction; ECP, extracorporeal photopheresis; eMIT, Drugs and Pharmaceutical Electronic Market Information Tool; PSSRU, Personal Social Services Research Unit; RAS, restrictive allograft syndrome; RR, relative risk.

### Health State Utilities

A targeted literature search was conducted for utility values. Base-case utility values were based on Esquinas et al,^14^ who reported utility values for chronic obstructive pulmonary disease for a variety of patient characteristics. These characteristics were reviewed, with FEV_1_ levels and the Hospital Anxiety and Depression Scale used to define utilities for stable FEV_1_ and steep decline in FEV_1_. The utility for moderate decline an average of stable and steep decline utilities.

### Unit Costs

Therakos Ltd provided a unit cost for ECP of £895 purchased or £877 rented. No UK-specific cost estimates by CLAD stage were identified. The cost of hospitalizations for each CLAD stage was calculated by multiplying the probability of hospitalization by the average cost of a hospitalization. The clinical expert (A.F.) expected hospitalizations to be 7 to 10 days on average. Using routine NHS cost data,^15^ we used “Currency code – DZ22M – Unspecified Acute Lower Respiratory Infection without Interventions, with CC score 13+,” giving a base case cost per hospitalization of £3756.

The medication resource use was informed by the E-CLAD UK trial protocol.^8,12^ In conversation with a clinical expert (A.F.), we established a typical dose for each medicine. This information was used to derive cost per 6-month period by combining least costly unit cost data obtained from the British National Formulary^16^ or the Electronic Market Information Tool.^17^

### Analyses

Population and treatment scenario analyses were conducted to understand how the incremental cost-effectiveness ratio (ICER) varied across scenarios. Parameter scenario and sensitivity analyses were conducted to identify the parameter values and assumptions that had the greatest impact on the ICER. A reference population and treatment protocol and base-case parameter values by population were defined so that the variation in ICER estimates across different populations, treatment protocols, and parameter values could be evaluated.

The reference population was all patients with confirmed CLAD as defined by Verleden et al,^5^ where 65% of patients are diagnosed in CLAD stage 1, 24% in CLAD stage 2, and 11% in CLAD stage 3.^18^ The following populations were compared with the reference population: bronchiolitis obliterans syndrome, restrictive allograft syndrome, CLAD stage 1, CLAD stage 2, and CLAD stage 3. Alternative scenarios included 75% diagnosed in CLAD stage 1 with fewer patients diagnosed in later stages and 55% diagnosed in CLAD stage 1 with more patients diagnosed in later stages. The model requires all patients to be in either CLAD 1, CLAD 2, or CLAD 3 at entry; and it allows the modeler to enter their own distribution.

In alternative scenarios, ECP is provided for only 6 months or provided indefinitely. In the 6-month scenario, a patient receives 9 cycles of ECP with each cycle, including 2 individual treatments (ie, 18 treatments). This aligns with the ECP protocol in the E-CLAD UK trial.^8,12^ The impact of changes in the waning effect was also explored. In the 6- and 12-month treatment scenarios, a waning period of 0 years (immediate loss of effectiveness), a waning period of 5 years, and no waning were explored. The intervention and population scenarios are described in **Table 2**. In addition, a 6-month treatment with a 1-year waning period, and 12-month treatment with 2- to 4-year waning scenarios were run to evaluate how much additional effectiveness duration is required to compensate for the additional cost of treatment.

**Table 2.** Population and Intervention Scenarios

**Analysis Name**	**Analysis Description**
Base case	12 months ECP; CLAD population
A: BOS	12 months ECP; BOS population
B: RAS	12 months ECP; RAS population
C: 4 ECP cycles/year indefinitely	Indefinite ECP; 4 ECP cycles/year from 2nd year; remain on ECP if initially responder; CLAD population
D: Stop ECP if rate of FEV_1_ decline becomes steep	Indefinite ECP; 4 ECP cycles/year from 2nd year; stop ECP if rate of FEV_1_ decline becomes steep; CLAD population
E: 2 ECP cycles/year indefinitely	Indefinite ECP; 4 ECP cycles/year from 2nd year; remain on ECP if initially responder; CLAD population
F: 6 months ECP	6 months ECP; CLAD population
G: CLAD 1	12 months ECP; CLAD 1 population
H: CLAD 2	12 months ECP; CLAD 2 population
I: CLAD 3	12 months ECP; CLAD 3 population

Abbreviations: BOS, bronchiolitis obliterans syndrome; CLAD, chronic lung allograft dysfunction; ECP, extracorporeal photopheresis; FEV_1_, forced expiratory volume in 1 second; RAS, restrictive allograft syndrome.

High and low parameter values were included in deterministic sensitivity analyses. A list of all parameter sensitivity and scenario analyses is reported in the **Supplementary Material**. While an implicit estimate of the relative risk of response for ECP vs standard care of 2.63 was included in the model, this estimate is associated with a high risk of bias. In this early economic model, one of the outcomes of interest is how effective ECP needs to be for it to be cost-effective. A threshold analysis was conducted where the relative risk of response for ECP vs standard care was varied until the ICER was £20 000/QALY. This was repeated for different threshold ICERs.

## RESULTS

**Figure 2.**
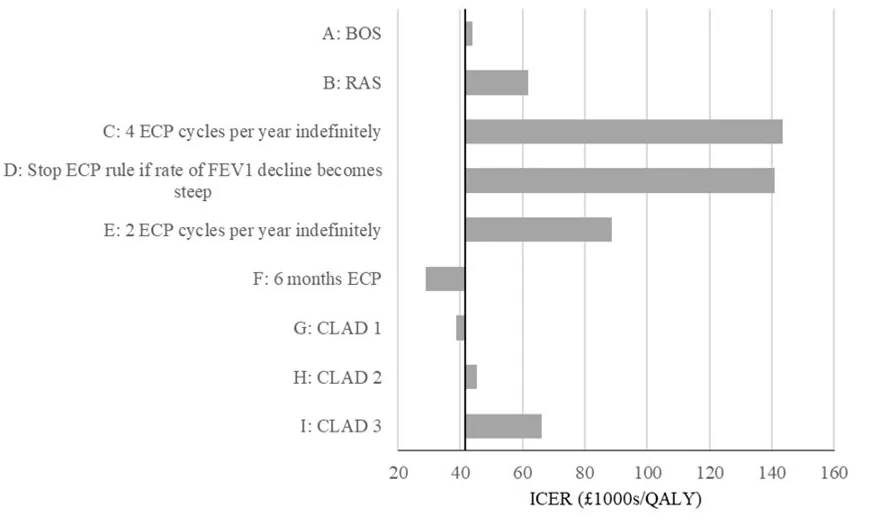
Tornado Diagram of the Population and Intervention Scenario Results Abbreviations: BOS, bronchiolitis obliterans syndrome; CLAD, chronic lung allograft dysfunction; ECP, extracorporeal photopheresis; FEV1, forced expiratory volume in 1 second; ICER, incremental-cost-effectiveness ratio; QALY, quality-adjusted life-year; RAS, restrictive allograft syndrome.

The ICER results for the alternative population and intervention analyses described in **Table 2** are presented in **Figure 2**. The vertical line represents the base case ICER for the reference population and treatment protocol (£41 470). The endpoint of the horizontal bars farthest from the vertical line represent the ICER of the alternative scenario. For example, the ICER for the restrictive allograft syndrome population was £61 763. The base case should be considered just one of many alternative, plausible scenarios.

**Figure 3.**
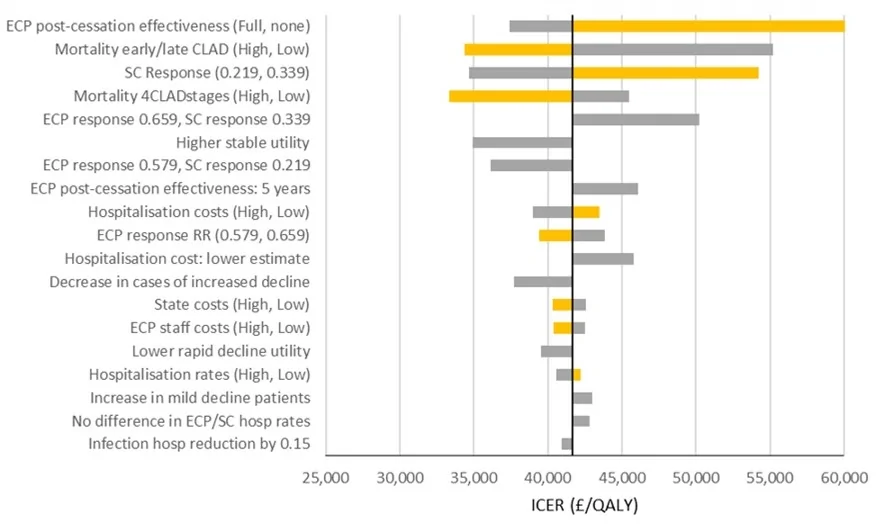
Tornado Diagram of Parameter Uncertainty Scenario Results Abbreviations: CLAD, chronic lung allograft dysfunction; ECP, extracorporeal photopheresis; ICER, incremental cost-effectiveness ratio; QALY, quality-adjusted life-year; RR, relative risk; SC, standard care.

The ICER results for the alternative parameter scenario and sensitivity analyses are presented in **Figure 3**. The gray bar represents the ICER that corresponds with the parameter value for the left value in the brackets in the analysis description or the single analysis if there is only one stated in the description. The amber bar corresponds to the ICER estimated using the right value in the brackets. The horizontal axis is truncated at £60 000/QALY. When it is assumed that the likelihood of worsening lung function decline is the same as with standard care for ECP after treatment cessation, the ICER is £88 278/QALY. The results of different treatment duration and waning scenarios are presented in **Table 3**.

**Table 3.** Survival Curves for ECP and Standard Care in the Base Case

	**Cost (£)**	**Incremental Cost**	**QALYs**	**Incremental QALYs**	**Life-Years**	**Incremental Life-Years**	**ICER (£)**
Standard care	76 137		2.360		5.18		
ECP	105 674	£29 537	3.072	0.712	6.14	0.962	41 470

Abbreviations: ECP, extracorporeal photopheresis; ICER, incremental cost-effectiveness ratio; QALY, quality-adjusted life-year.

With all other parameters set to their base-case values, a threshold analysis for the relative risk revealed that the threshold relative risk (RR) of response for ECP compared with standard care was 5 for a cost-effectiveness threshold of £30 000; 2.18 for a cost-effectiveness threshold of £50 000; and 1.52 for a cost-effectiveness threshold of £100 000. The RR increases exponentially as the ICER falls. This is determined by both the mathematical properties of multiplying the standard care risk of the condition worsening by the inverse of the RR, and by the standard care risk.

## DISCUSSION

The early economic evaluation model can evaluate the cost-effectiveness of interventions to treat CLAD such as ECP. While there are economic models of lung transplants^19^ and immunosuppression therapies following lung transplantation,^20^ these are not relevant for people with definitive CLAD. This is an early economic model with highly uncertain model parameters and assumptions. The purpose is to provide plausible ranges of cost-effectiveness, understand the relative importance of different factors toward cost-effectiveness, and create a model that can be adapted as new evidence emerges.

Our model can estimate threshold values for specific parameters that result in a predefined threshold ICER value for an intervention. These threshold values can be help justify collection and analysis of data from ongoing and future studies, which can subsequently be used to update model parameter values.

As an early economic model, it should be recognized that there is very limited evidence on the effectiveness of ECP vs current practice. The response probabilities were obtained from retrospective cohort studies, and the implicit relative effectiveness estimate is at high risk of bias, the magnitude and direction of which is unknown. The uncertainty associated with this risk of bias could not be modeled. Threshold analysis was done to estimate the RR required to obtain different cost-effectiveness thresholds. The question, “Is it plausible that the intervention could be X times better than standard care?” can then be posed. If it is not plausible, then this suggests that future research on the intervention in its current form is unlikely to be worthwhile. These threshold results are based on the base-case assumption of ECP continuing to reduce the likelihood that lung function worsens compared with standard care for a period of 10 years with the effectiveness declining to zero over that time. If the treatment waning period were shorter, then the RR estimates would need to be greater.

The RR of response is modeled to reduce the likelihood that the condition worsens while receiving ECP. How likely the condition is to worsen while on ECP is unknown, and clinical studies with a sufficient follow-up period are required. The use of separate cohort studies for the response probabilities for ECP and standard care explains why the ICERs for both bronchiolitis obliterans syndrome and restrictive allograft syndrome are higher than that for CLAD. Better data on both from a well-matched cohort may change this picture. The earlier the CLAD stage that ECP is started, the lower the ICER as more patients are prevented from progressing to more severe CLAD stages.

The duration of ECP treatment and the assumptions around continuing effectiveness of ECP after cessation of treatment also had a significant effect on the ICER. Other things being equal, increasing the duration of ECP treatment increases the ICER (additional cost with no additional benefit), and increasing the duration of waning reduced the ICER (additional benefit at no extra cost). ECP is modeled to reduce the likelihood of worsening lung function decline over time for patients who initially respond, not to eliminate it; there is considerable uncertainty in this area. Increasing the waning period compensated for increases in the duration of treatment over short waning periods (1-4 years) only because median survival is modeled to be much shorter than the 5- and 10-year waning period scenarios.

Mortality rates have a large impact on the ICER. When early CLAD stage mortality rates are relatively high (ie, there is a smaller difference in mortality rates between early and late CLAD stages), the ICER increases considerably. This is because the benefit of delaying progression to more severe CLAD stages is smaller. When early CLAD stage mortality rates are relatively low, the reverse is true.

Changing the response probability of standard care (which changes the FEV_1_ decline category transition probabilities for standard care) also affects the ICER, as this effectively changes the relative effectiveness of ECP vs standard care. Within the model, response probabilities for both standard care and ECP were varied between the 95% CI limits of the response estimates. However, as response estimates for ECP had a narrower 95% CI than the corresponding data for standard care, the impact of varying ECP response rates on the ICER was much less than varying standard care response rates. Thus, this finding from the sensitivity analysis is an artifact of the sparse data available for modeling.

As there is no UK clinical guideline on how best to treat lung transplantation patients, there is likely to be practice variation across centers. Although this initial costing of medications is based on what may be seen in lung transplantation patients, based on the authors’ experience, medication costs may vary as much within a country as it does between countries. Further data on actual medications used would allow the impact of medication costs and variations in medication costs to be explored. At present, medication costs are estimated to be £9446 per year per person based on medication utilization provided by expert advice and published unit cost estimates. The E-CLAD UK trial may provide better estimates. These, however, are not key determinants of the ICER. Key determinants include the effectiveness of ECP after treatment cessation (whether there is a temporary or permanent therapeutic benefit), the response rate for standard care (including the rate of lung function decline over time), and mortality rates.

For the reference population and treatment protocol, the ICER estimates range from £33 343 to £88 278. This is not the full range of possible ICER values as multiple factors together may result in a wider variation in ICER values and the full range of possible RRs of response (which is unknown) is not reflected, but our estimates represent a current best guess. Whether these are judged to be sufficiently promising depends upon what a decision-maker may be willing to pay for a QALY. In England, for many healthcare technologies, NICE recommends an ICER of £25 000/QALY to £35 000/QALY for decision making. NICE allows higher thresholds for technologies that meet the criteria of highly specialized technologies.^21^ The four eligibility criteria are as follows:

1. The condition is very rare (prevalence of <1 per 50 000 in England).
2. No more than 300 people in England are eligible for the technology in its licensed indication and no more than 500 across all its indications.
3. The condition the technology is indicated for significantly shortens life or severely impairs quality of life.
4. There are no satisfactory treatment options, or the technology is likely to offer significant additional benefit over existing treatment options.

If these criteria are met, the ICER can be £100 000/QALY or higher. These criteria almost apply to the use of ECP for CLAD; ECP is currently used in fewer than 500 cases across indications per year in England, but this would rise if it were recommended for CLAD patients.^22^

The population for whom ECP would be most effective and cost-effective is still unknown. The evidence from Benazzo et al^9^ (for brochiolitis obliterans syndrome) and Gautschi et al^23^ (for CLAD) both report a survival benefit for ECP. However, ongoing and future trials are needed to determine how ECP should be delivered and to whom.

### Areas for Further Work

The early economic model indicates that effectiveness estimates for ECP in a CLAD population from a well-designed and conducted trial are required and should be analyzed using best practices. Such evidence needs to be longitudinal with sufficient follow-up to show how FEV_1_ changes over time both with and without ECP. Any study that can provide evidence on mortality rates by CLAD stage and by rate of decline in FEV_1_ also needs to be sufficiently long to provide mature data on mortality rates by CLAD stage and by rate of decline in FEV_1_. Data maturity can be defined in relation to the percentage of patients being followed up for a target period of *X* years or until an event occurs for the statistical analysis of difference in survival.^24^ However, for economic modeling, when the percentage of patients experiencing an event within 10 years is expected to be high, then the closer the percentage of patients experiencing the event is to 70% the better, with 50% being much better than 30%.^25^ There is also little published evidence on hospitalization rates and health utilities by CLAD stage and by rate of decline in FEV_1_. The model can also be refined based on new evidence on the relative importance of different CLAD stages and rates of decline in FEV_1_ to cost, QoL and mortality outcomes.

The E-CLAD UK trial and its long-term follow-up study will provide evidence in this respect, but data from other countries would allow both precision and heterogeneity to be explored.^8,12^

As an early economic evaluation model, our analysis has omitted a probabilistic sensitivity analysis, as would normally be expected.^11^ This is because there are a great number of uncertain assumptions and highly uncertain parameter values, due to either a complete lack of published evidence or a considerable risk of bias in the evidence. In both cases, it is inappropriate to specify probability distributions for parameter values, as there is no valid published evidence to inform those distributions. As better, more rigorous data become available, the economic evaluation model should be updated to include a probabilistic sensitivity analysis to explore the impact of parameter uncertainty on our results and clarify where important evidence gaps remain.

## CONCLUSION

An early economic model has been developed and populated with parameter values based on the best available evidence. Rate of decline in FEV_1_ is the driver of clinical decision and patient outcomes. A wide range of ICER estimates are plausible for ECP vs standard care. While many of these estimates are above cost-effectiveness thresholds that NICE recommends for healthcare technologies, ECP comes close to qualifying as a highly specialized technology, for which NICE accepts much higher cost-effectiveness thresholds. Clinical studies such as the E-CLAD UK trial are in progress or planned. As new evidence for the model parameters emerges, the model can be updated.

### Disclosures

S.R., L.V., D.C., and A.J.F. works or worked for Newcastle University and Newcastle University received funding from Therakos Ltd to undertake this work.

## Supporting information

Online Supplementary Material

## References

[1] Statista Estimated number of lung transplantations worldwide in 2022, by region.

[2] Chambers D.C., Cherikh W.S., Goldfarb S.B.. (2018). The International Thoracic Organ Transplant Registry of the International Society for Heart and Lung Transplantation: Thirty-fifth Adult Lung and Heart-Lung Transplant Report–2018. J Heart Lung Transplant.

[3] NHS Blood and Transplant (2024). Annual Report on Lung Transplantation 2023/24.

[4] Agence de la Biomédecine Rapport médical et scientifique 2023.

[5] Verleden G. M., Glanville A. R., Lease E. D.. (2019). Chronic lung allograft dysfunction: definition, diagnostic criteria, and approaches to treatment–a consensus report from the Pulmonary Council of the ISHLT. J Heart Lung Transplant.

[6] Meyer K. C., Raghu G., Verleden G. M.. (2014). An international ISHLT/ATS/ERS clinical practice guideline: diagnosis and management of bronchiolitis obliterans syndrome. Eur Respir J.

[7] Halloran K., Aversa M., Tinckam K.. (2018). Comprehensive outcomes after lung retransplantation: a single-center review. Clin Transplant.

[8] Fisher A. J., White M., Goudie N.. (2024). Extracorporeal photopheresis (ECP) in the treatment of chronic lung allograft dysfunction (CLAD): a prospective, multicentre, open-label, randomised controlled trial studying the addition of ECP to standard care in the treatment of bilateral lung transplant patients with CLAD (E-CLAD UK). BMJ Open Respir Res.

[9] Benazzo A., Bagnera C., Ius F.. (2023). A European multi-center analysis of extracorporeal photopheresis as therapy for chronic lung allograft dysfunction. Transpl Int.

[10] Kim H., Goodall S., Liew D. (2021). The potential for early health economic modelling in health technology assessment and reimbursement decision-making. Int J Health Policy Manag.

[11] NICE (2023). NICE health technology evaluations: the manual.

[12] Fisher P. A. (2023). BMC.

[13] Todd J. L., Neely M. L., Finlen Copeland C. A.. (2019). Prognostic significance of early pulmonary function changes after onset of chronic lung allograft dysfunction. J Heart Lung Transplant.

[14] Esquinas C., Ramon M. A., Nunez A.. (2020). Correlation between disease severity factors and EQ-5D utilities in chronic obstructive pulmonary disease. Qual Life Res.

[15] England N. (2023). 2021/22 National Cost Collection Data Publication.

[16] NICE (2023). British National Formulary (BNF).

[17] GOV.UK Drugs and pharmaceutical electronic market infromation tool (eMIT).

[18] Nykanen A., Raivio P., Perakyla L.. (2020). Incidence and impact of chronic lung allograft dysfunction after lung transplantation - single-center 14-year experience. Scand Cardiovasc J.

[19] Peel J. K., Keshavjee S., Krahn M.. (2021). Economic evaluations and costing studies of lung transplantation: a scoping review. J Heart Lung Transplant.

[20] Sharples L. D., Taylor G. J., Karnon J.. (2001). A model for analyzing the cost of the main clinical events after lung transplantation. J Heart Lung Transplant.

[21] NICE (2024). NICE-Wide Topic Prioritisation: The Manual.

[22] Therakos (2024). Therakos internal data.

[23] Gautschi F., Vogelmann T., Ortmanns G.. (2024). Early extracorporeal photopheresis treatment is associated with better survival in patients with chronic or recurrent acute lung allograft dysfunction. J Clin Apher.

[24] Gebski V., Gares V., Gibbs E.. (2018). Data maturity and follow-up in time-to-event analyses. Int J Epidemiol.

[25] Roze S., Bertrand N., Eberst L.. (2023). Projecting overall survival in health-economic models: uncertainty and maturity of data. Curr Med Res Opin.

