# Supplementary material for: Early Economic Model of Extracorporeal Photopheresis and Other Treatments of Chronic Lung Allograft Dysfunction After Lung Transplantation
Source: J Health Econ Outcomes Res. 2026 Sep 22;13(2):104–11. doi: 10.36469/001c.163450 (PMC13613274; doi:10.36469/001c.163450)
Supplement: Supplementary file 1 [file jheor_2026_13_2_163450_367652.pdf]

Journal of Health Economics and Outcomes Research

Online Supplementary Material

Early Economic Model of Extracorporeal Photopheresis and Other Treatments of Chronic Lung Allograft Dysfunction After Lung Transplantation. *JHEOR*. 2026;13(2):104-111. doi:10.36469/jheor.2026.163450

1. **Methods Associated with NICE Guidance** 1
2. **Model Development** 1
3. **Lung Function Decline Category Definitions** 2
4. **Standard Care Transition Probabilities** 3
5. **Response Rates** 6
6. **Mortality** 6
7. **Standard Care Hospitalization Rates** 7
8. **Utility Values** 8
9. **Medication Costs** 10
10. **Scenario Analyses** 11
11. **Survival and Lung Function Model Prediction Curves** 12
12. **Clinical Care Pathway for Model** 13

This supplementary material has been provided by the authors to give readers additional information about their work.

This is an open-access article distributed under the terms of the Creative Commons Attribution 4.0 International License (CCBY-4.0). View this license's legal deed at http://creativecommons.org/licenses/by/4.0 and legal code at http://creativecommons.org/licenses/by/4.0/legalcode for more information.

Supplementary Material

Contents

1. Methods associated with NICE guidance
2. Model development
3. Lung function decline category definitions
4. Standard care transition probabilities
5. Response rates
6. Mortality
7. Standard care hospitalization rates
8. Utility values
9. Medication costs
10. Scenario analyses
11. Survival and lung function model prediction curves
12. Clinical care pathway for model

1. Methods used consistent with NICE guidance

The analysis presented in this case is undertaken from a National Health Service (NHS) and Personal Social Services (PSS) perspective. The measure of benefit was quality-adjusted life-years (QALYs). An annual discount rate of 3.5% was used. A 25-year time horizon was chosen as the model predictions were that less than 1% of people receiving ECP are alive after 25 years.

2. Model development

The model development process first followed an initial model design phase, and then second a model refinement, and analysis phase. The process is presented in Supplementary Figure 1.

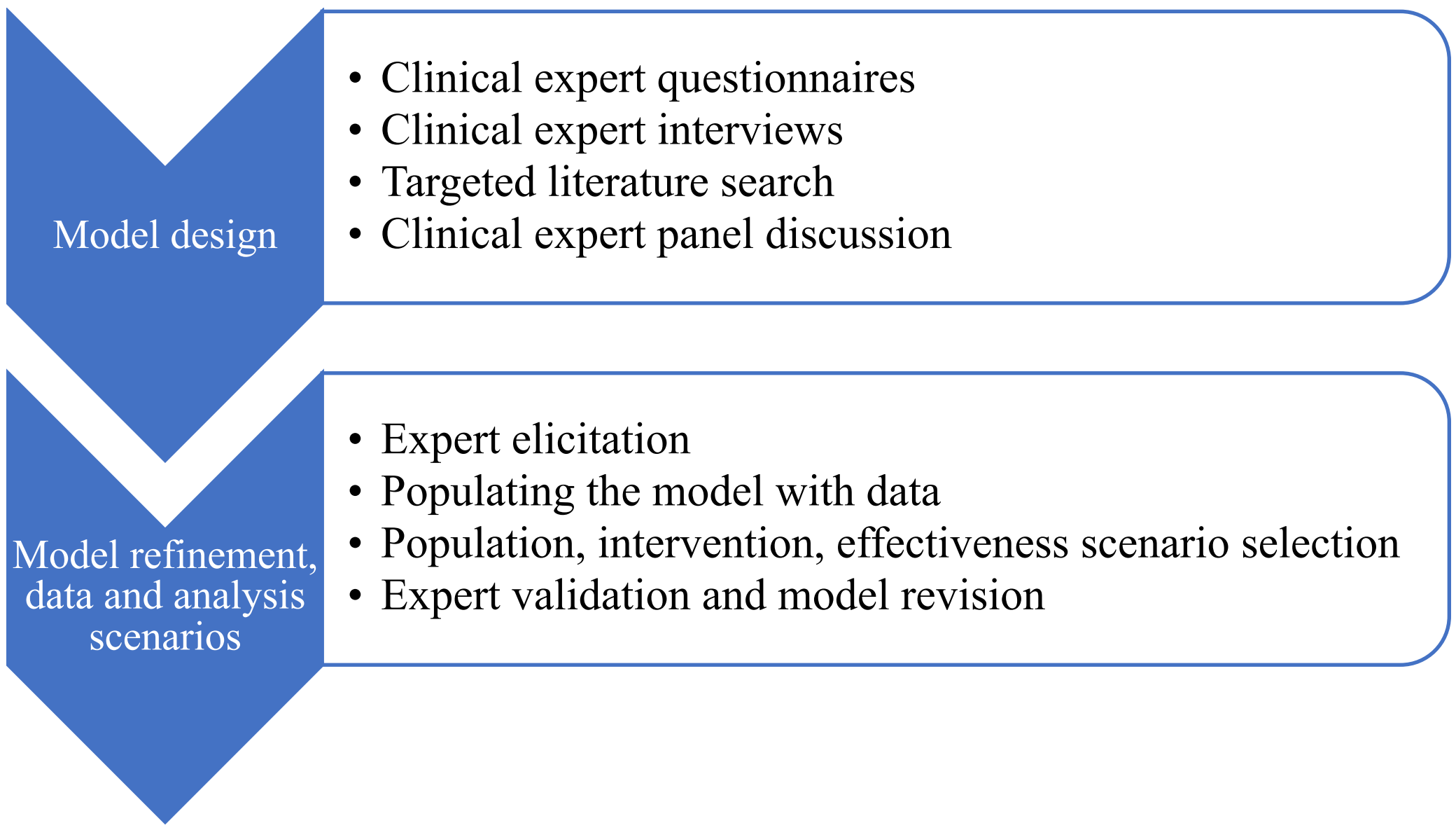

Supplementary Figure 1: Model development stages

3. Lung function decline categories

For each of the 5 categories of FEV1 decline, there is a tunnel state to represent the movement to the category from another FEV1 decline category. Patients do not stay in these tunnel states (NR: new rate); they either move to another FEV1 decline category in the next cycle or they move to a state representing the patient remaining in that FEV1 decline category (OR: ongoing rate). Patients in OR states can remain in the state in the next cycle. The distinction between the tunnel states (NR) and the OR states introduced the possibility of there being some stability within each FEV decline category by making it more likely to remain in the same FEV1 decline category if they had been in that category for 2 cycles.

FEV1 decline values used in the model are presented in Supplementary Table 1. For example, OR7 is the state where people with CLAD remain in the $-10\% \leq X < -3\%$ FEV1 decline category. The actual reduction in FEV1 over 6 months in the model is the assumed change

presented in Supplementary Table 1. In the base case, the high values are used. But low values can also be implemented in the model.

Supplementary Table 1: Number codes for %FEV1 decline categories and assumed change in FEV1 in the model

Change in FEV1 categories (measured every 6 months)	Reference number used in model	Assumed change in FEV1 over 6 months in model	
		**High**	**Low**
X < -30% FEV1	35	-35%	-25%
-30% ≤ X < -10% FEV1	20	-20%	-15%
-10% ≤ X < -3% FEV1	7	-6.5%**	-6.5%
-3% ≤ X < 3% FEV1	0	0%	0%
X ≥ 3% FEV1	3	6.5%*	6.5%

*Only applies for an ongoing rate (OR3). New rates of >3% are assumed to have 0% increase. This avoids the possibility of a small % of patients with forever increasing FEV1 levels in the model. An increase of 10% for OR3 should compensate for this.

**Does not apply for transitions from NR3. This compensates for no increase in FEV1 for transitions to NR3.

4. Standard care transition probabilities

Due to the lack of published evidence to inform lung function decline the economic model, clinical expert elicitation was conducted.

Experts were asked to describe common patterns of lung function decline, e.g. decline then plateau; decline then plateau then decline (stepped); etc. They were then asked to estimate roughly the percentage of patients that fall into each category. Using some judgement of the modeller (SR), transition probabilities between FEV1 decline categories were derived.

A variety of possible lung function decline trajectories for survivors were identified, examples presented in Supplementary Figure 2. Roughly 41% of trajectories were assumed to be stepped (decline, plateau, decline). 14% were assumed to be continuous steep decline. 26% were assumed to be continuous moderate decline. 19% were assumed to be either mild decline followed by a plateau or an immediate small increase followed by a plateau or a mild decline. Changes in % FEV1 over 6-month periods were converted to transition probabilities between lung function decline categories, using some modelling judgement by the principal modeler (SR). Transition probabilities were assumed to be constant over time, apart from the exception that within the first 2-years there was a possibility that patients could improve their FEV1 level. Transition probabilities were further edited so that the model predicted the proportion of the patients with stable lung function to be closer to clinical expert opinion discussed in the expert validation meeting. Fundamentally, there is considerable uncertainty in the transition probabilities; therefore, the standard care transition probabilities and therefore the lung function trajectories were varied in scenario analysis to explore the effect on the ICER estimates.

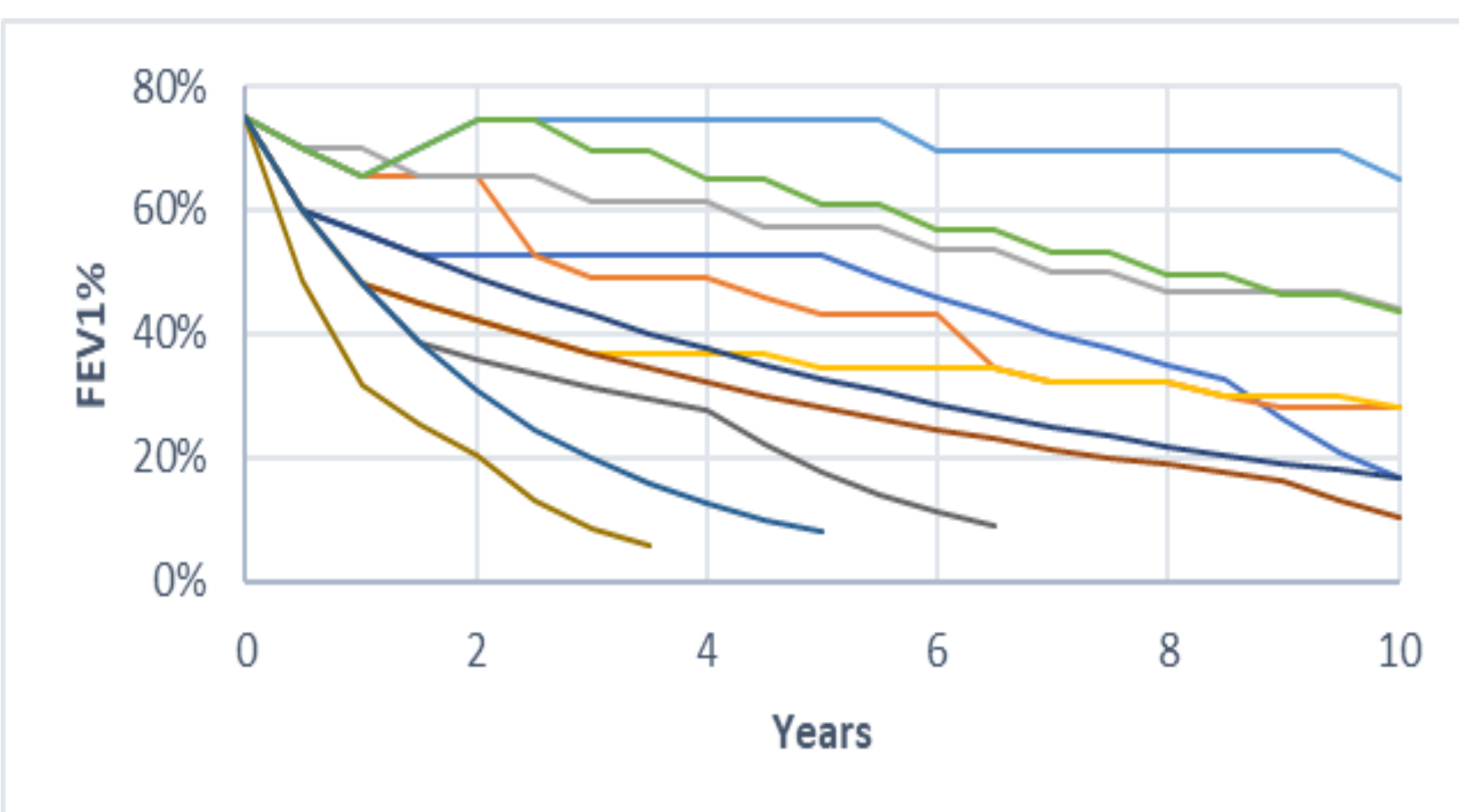

Supplementary Figure 2: Example lung function decline trajectories

Transition probabilities were set for the initial 6 months, the period 6 to 24 months, and beyond 24 months. An increase in FEV1 was assumed to only be possible during the first 2 years. The base case transition probabilities are presented in Supplementary Tables 2-6.

Supplementary Table 2: Initial 6-month transition probabilities

	**0.065**	**0**	**-0.05**	**-0.125**	**-0.25**
**0.065**	0.66	0.34	0	0	0
**0**	0.3	0.65	0.05	0	0
**-0.05**	0.1	0.2	0.7	0	0
**-0.125**	0.05	0.13	0.19	0.63	0
**-0.25**	0	0	0.1	0.2	0.7

Supplementary Table 3: 6-24 months, New Rate transition probabilities

	**0.065**	**0**	**-0.05**	**-0.125**	**-0.25**
**0.065**	1	0	0	0	0
**0**	0	0.55	0.15	0.3	0
**-0.05**	0	0.22	0.05	0.73	0.00
**-0.125**	0	0.43	0.12	0.38	0.07
**-0.25**	0	0.00	0.00	0.41	0.59

Supplementary Table 4: 6-24 months, Ongoing Rate transition probabilities

	**0.065**	**0**	**-0.05**	**-0.125**	**-0.25**
**0.065**	1	0	0	0	0
**0**	0	0.4	0.15	0.45	0
**-0.05**	0	0.05	0.5	0.45	0
**-0.125**	0	0.13	0.35	0.42	0.10
**-0.25**	0	0.00	0.00	0.71	0.29

Supplementary Table 5: >24 months, New Rate transition probabilities

	**0.065**	**0**	**-0.05**	**-0.125**	**-0.25**
**0.065**	0	1	0	0	0
**0**	0	0.55	0.15	0.3	0
**-0.05**	0	0.22	0.05	0.73	0.00
**-0.125**	0	0.43	0.12	0.38	0.07
**-0.25**	0	0.00	0.00	0.41	0.59

Supplementary Table 6: >24 months, Ongoing Rate transition probabilities

	**0.065**	**0**	**-0.05**	**-0.125**	**-0.25**
**0.065**	0	1	0	0	0
**0**	0	0.4	0.15	0.45	0
**-0.05**	0	0.05	0.5	0.45	0
**-0.125**	0	0.13	0.35	0.42	0.10
**-0.25**	0	0.00	0.00	0.71	0.29

5. Response rates

For ECP, the response probability for CLAD was reported in Benazzo et al 2024.[7] In the same study, other related but not identical information was reported for BOS and RAS were used to derive the BOS and RAS probabilities using a weighted average approach. The response probabilities for BOS and RAS for standard care were obtained from Todd et al. 2019,[11] using reported event data and sample sizes.

6. Mortality

Supplementary Table 7: Mortality probability selection names and their associated publications

Mortality probability selection names	Publication probability calibrated against	Treatment used in study
Base case	Survival prediction in-between Todd 2014[12] and Nykanen 2020[13]	
Todd4	Todd 2014[12]	Standard care
Nykanen4	Nykanen 2020[13]	Standard care
Base case 2	Survival prediction in-between Gautschi 2024[14] and Benazzo 2024[7]	
Todd2	Gautschi 2024[14]	ECP
Nykanen2	Benazzo 2024[7]	ECP

Supplementary Table 8: 6-month mortality probabilities by CLAD stage and FEV1 decline rate

	Base case	Todd4	Nykanen4	Base case2	Todd2	Nykanen2
CLAD0	0.024	0.029	0.010	0.04	0.07	0.02
CLAD1low	0.024	0.029	0.010	0.04	0.07	0.02
CLAD1mod	0.078	0.117	0.030	0.05	0.10	0.02
CLAD1sev	0.087	0.193	0.053	0.08	0.13	0.05
CLAD2low	0.047	0.040	0.012	0.04	0.07	0.02
CLAD2mod	0.101	0.128	0.032	0.05	0.10	0.02
CLAD2sev	0.110	0.204	0.054	0.08	0.13	0.05
CLAD3low	0.071	0.081	0.023	0.10	0.15	0.07
CLAD3mod	0.125	0.168	0.043	0.11	0.18	0.08
CLAD3sev	0.134	0.245	0.065	0.14	0.22	0.10
CLAD4low	0.100	0.101	0.076	0.10	0.15	0.07
CLAD4mod	0.116	0.218	0.126	0.11	0.18	0.08
CLAD4sev	0.162	0.264	0.129	0.14	0.22	0.10

Supplementary Table 9: Hazard ratios of mortality for BOS and RAS vs CLAD

Comparison	Hazard ratio of mortality	Source
RAS vs BOS	2.73, 95% CI: 1.86 to 4.04	Todd et al. 2019[11]
BOS vs CLAD	0.82, 95% CI: 0.72 to 0.90	Derived from Todd et al. 2019[11]
RAS vs CLAD	2.23, 95% CI: 1.67 to 2.90	Derived from Todd et al. 2019[11]

7. Standard care hospitalization rates

Standard care hospitalization probabilities by CLAD stage per year were obtained from a clinical expert. These are reported in Supplementary Table 10. For ECP, it is assumed that the probabilities are 80% of the probabilities for standard care. There is no published data to support this. No difference in probabilities was explored in a scenario analysis.

Supplementary Table 10: Annual probability of hospitalization for standard care

CLAD 1	CLAD 2	CLAD 3	CLAD 4
0.2	0.3	0.4	0.7

8. Utility values

A targeted literature search was conducted for utility values. Base case utility values were based on Esquinas et al. 2020,[15] which reported utility values for chronic obstructive pulmonary disease for a variety of patient characteristics. All of these characteristics were reviewed, with FEV1 levels and HADS anxiety particularly informing stable FEV1 and steep decline with moderate decline an average of stable and steep decline utilities. The base case utilities are reported in Supplementary Table 11.

Supplementary Table 11: Base case utility values

FEV1FEV1 decline	CLAD1	CLAD2	CLAD3	CLAD4
Stable	0.7	0.7	0.66	0.6
Moderate decline	0.56	0.56	0.516	0.45
Steep decline	0.42	0.42	0.372	0.3

A higher stable utility scenario was produced based on Kim et al. 2014[16] which reported higher utility values for GOLD criteria categories. These were capped at 0.8, the average EQ-5D utility index score for people aged 55-64 in the general population.[17] These utilities are reported in Supplementary Table 12.

Supplementary Table 12: Higher stable utility scenario

FEV1FEV1 decline	CLAD1	CLAD2	CLAD3	CLAD4
Stable	0.8	0.8	0.724	0.61
Moderate decline	0.61	0.61	0.548	0.455
Steep decline	0.42	0.42	0.372	0.3

A lower steep decline utility scenario was produced. The CLAD 1/steep decline utility was based on the EQ-5D-5L utility state 43324 (0.3) and the CLAD 4/steep decline utility was based the EQ-5D-5L utility state 43434 (0.215). These utilities are reported in Supplementary Table 13.

Supplementary Table 13: Lower steep decline utility scenario

FEV1FEV1 decline	CLAD1	CLAD2	CLAD3	CLAD4
Stable	0.7	0.7	0.66	0.6
Moderate decline	0.5	0.5	0.463	0.4075
Steep decline	0.3	0.3	0.266	0.215

A 'high stable' scenario was produced with higher CLAD 4 utility. These utilities are reported in Supplementary Table 14.

Supplementary Table 14: High stable scenario

FEV1FEV1 decline	CLAD1	CLAD2	CLAD3	CLAD4
Stable	0.8	0.8	0.814	0.7
Moderate decline	0.61	0.61	0.593	0.5
Steep decline	0.42	0.42	0.372	0.3

A high stable CLAD1 and low steep decline utility scenario is reported in Supplementary Table 15.

Supplementary Table 15: High stable CLAD 1 and low steep decline scenario

FEV1FEV1 decline	CLAD1	CLAD2	CLAD3	CLAD4
Stable	0.8	0.7	0.66	0.6
Moderate decline	0.55	0.5	0.463	0.4075
Steep decline	0.3	0.3	0.266	0.215

9. Medication costs

Medication unit costs are described in Supplementary Table 16. Medication sources included the BNF[19] and Emit.[20]

Supplementary Table 16: Medication unit costs

	Dosage size	Price	Pack size	Source
Maintenance Immunosuppression:				
Tacrolimus (Adoport 0.5mg)	500mcg	42.92	50	BNF
Cyclosporine (Neoral TM)	100mg	68.28	30	BNF
Prednisolone	5mg	0.85	28	BNF
Azathioprine	100mg	3.18	28	BNF
Methotrexate	2.5mg	1.62	28	BNF
MMF (Generic)	250mg	5.98	50	BNF
Anti-microbial prophylaxis (if indicated)				
co-trimoxazole	80mg/400mg	2.26	28	BNF
Valganciclovir	450mg	91.74	60	eMIT
Fluconazole	50mg	0.39	7	eMIT
Voriconazole	200mg	38.9	28	eMIT
Posaconazole	100mg	61.3	24	eMIT
Amphotericin B (Severe TX)	50mg	16.21	1	BNF
Inhaled colistin (Main one used)		204	30	BNF
Treatment of Gastro-oesophageal Reflux				
Esomeprazole	40mg	3.17	28	eMIT
Lansoprazole	30mg	1.12	28	BNF

10. Scenario analyses

Supplementary Table 17: Analyses exploring the impact of varying parameter values

	Scenario/sensitivity analyses	Description
1	ECP post-cessation effectiveness (Full, none)	Full ECP effectiveness continues beyond treatment cessation; No effect beyond
2	SC Response (0.219, 0.339)	Low and high response rates for standard care
3	Mortality 4CLADstages (High, Low)	Mortality rates assumed different for each CLAD stage; high (smaller differences in mortality rates between early CLAD stages and later CLAD stages), low (larger differences in mortality rates between early CLAD stages and later CLAD stages)
4	ECP response RR (0.579, 0.659)	Low and high response rates for ECP
5	Hospitalization cost: lower estimate	A lower hospitalization cost: short stay cost estimate
6	Hospitalization rates (High, Low)	Higher and lower hospitalization rates
7	ECP response 0.579, SC response 0.219	Lower response rates for both ECP and standard care
8	No difference in ECP/SC hosp rates	Hospitalization rates assumed to be the same for both ECP and standard care
9	Higher stable utility	The utility values for stable lung function assumed to be the same
10	Hospitalization costs (High, Low)	The cost of hospitalization is increased by 20% or reduced by 20%
11	ECP response 0.659, SC response 0.339	Higher response rates for both ECP and standard care
12	Lower rapid decline utility	Lower utility values for rapid decline in FEV1FEV1 states
13	State costs (High, Low)	The average cost of CLAD stage costs are increased by 20% or reduced by 20%
14	Increase in mild decline patients	Increase the likelihood of a mild decline in FEV1 instead of no decline
15	Decrease in cases of increased decline	This scenario reduces the probability that the lung function of a patients changes from stable to moderate to steep decline
16	ECP staff costs (High, Low)	ECP staff costs are increased by 20% or reduced by 10%
17	Infection hosp reduction by 0.15	To avoid discounting, the hospitalization probability by CLAD stage is reduced by 0.25 in base case; in this scenario, it is only reduced by 0.15
18	ECP post-cessation effectiveness: 5 years	Waning period after ECP cessation assumed to be 10 years in base case. This is reduced to 5 years in this scenario
19	Mortality early/late CLAD (High, Low)	Mortality rates assumed same for CLAD stages 1 and 2, and for CLAD stages 3 and 4; high (smaller differences in mortality rates between early CLAD stages and later CLAD stages), low (larger differences in mortality rates between early CLAD stages and later CLAD stages)

11. Survival and lung function model prediction curves

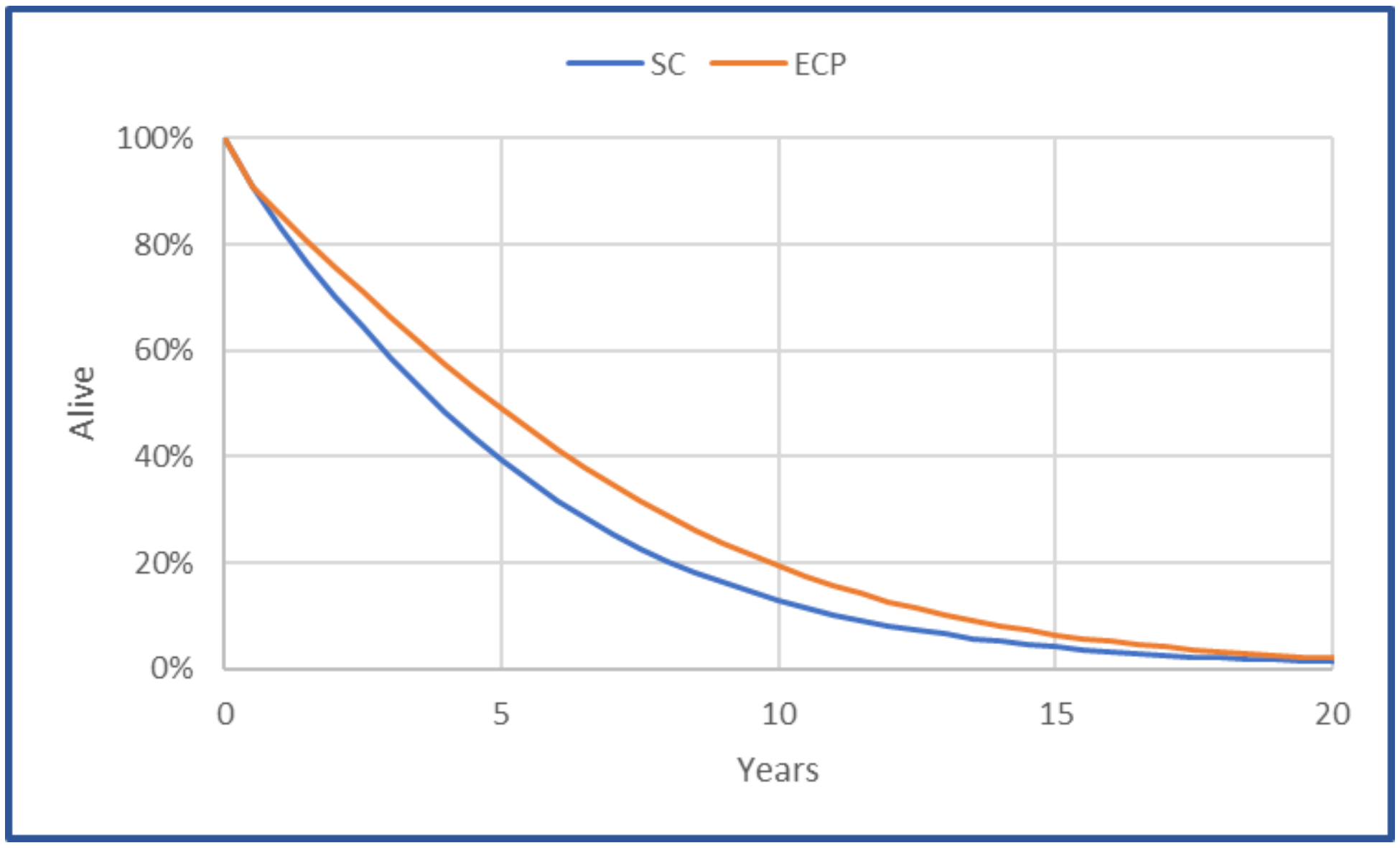

Figure 1: Survival curves for ECP and standard care (SC) in the base case

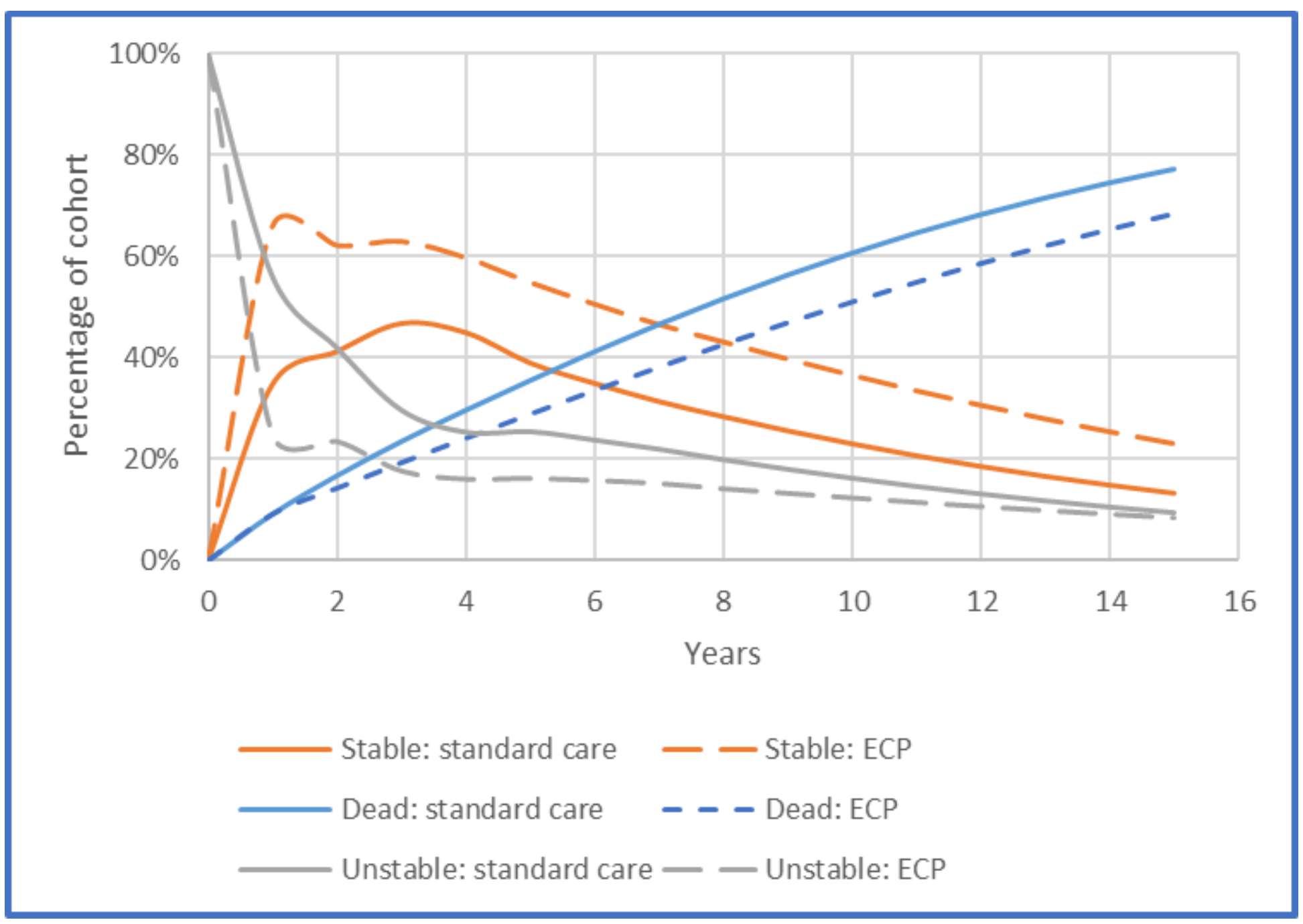

Figure 2: Percentage of cohort stable, unstable or dead for ECP and standard care

12. Clinical care pathway for model

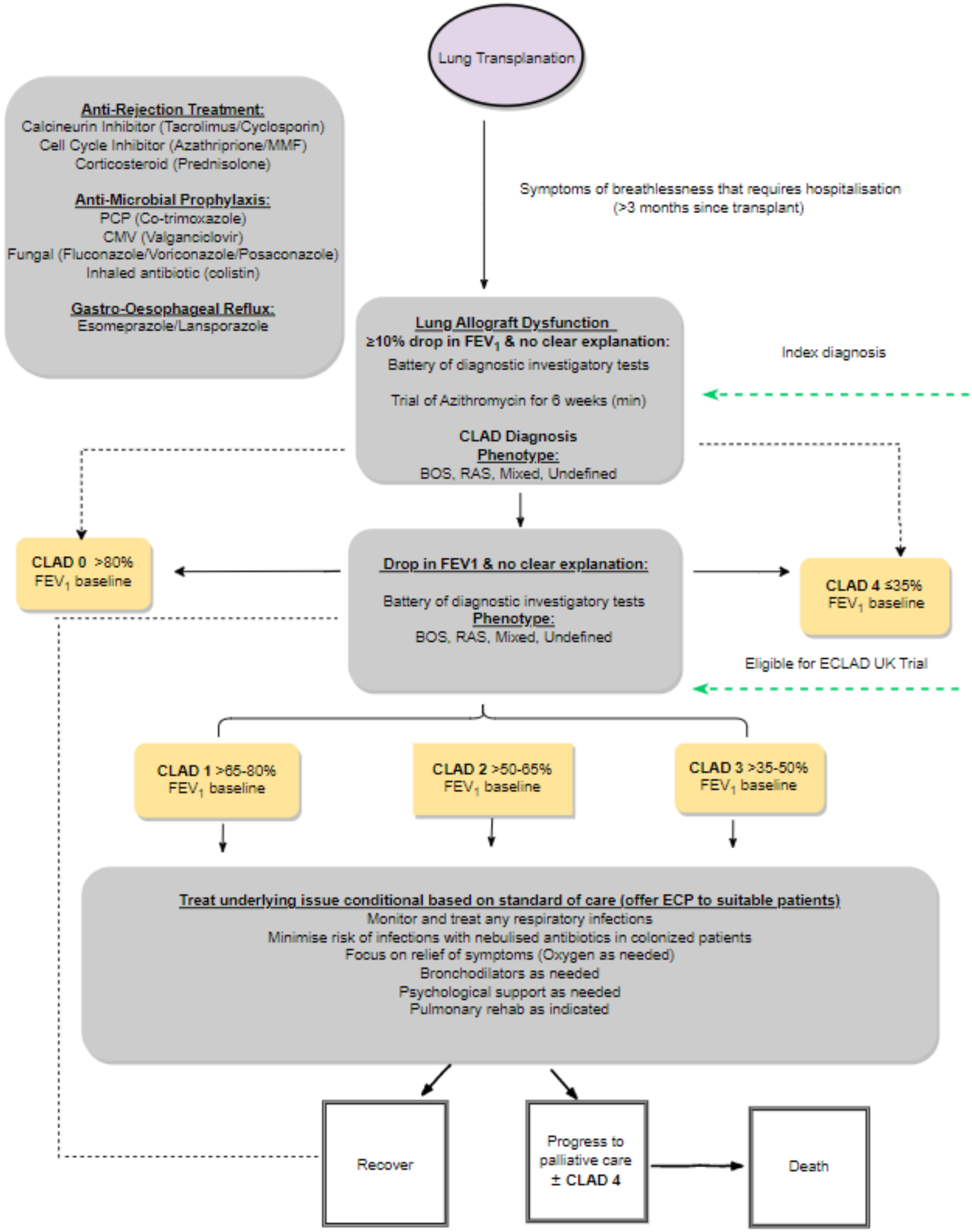

Figure 3: Basic conceptualization of the CLAD care pathway for an economic model
